# Fabrication and Characteristics of an nc-Si/c-Si Heterojunction MOSFETs Pressure Sensor

**DOI:** 10.3390/s120506369

**Published:** 2012-05-14

**Authors:** Xiaofeng Zhao, Dianzhong Wen, Gang Li

**Affiliations:** Key Laboratory of Electronics Engineering, College of Heilongjiang Province, Major Laboratories of Integrated Circuits, Heilongjiang University, Harbin 150080, China; E-Mails: zxf80310@126.com (X.Z.); lig8-78@msn.com (G.L.)

**Keywords:** nc-Si/c-Si heterojunction, MOSFETs pressure sensor, MEMS technology, CMOS process

## Abstract

A novel nc-Si/c-Si heterojunction MOSFETs pressure sensor is proposed in this paper, with four p-MOSFETs with nc-Si/c-Si heterojunction as source and drain. The four p-MOSFETs are designed and fabricated on a square silicon membrane by CMOS process and MEMS technology where channel resistances of the four nc-Si/c-Si heterojunction MOSFETs form a Wheatstone bridge. When the additional pressure is P, the nc-Si/c-Si heterojunction MOSFETs pressure sensor can measure this additional pressure P. The experimental results show that when the supply voltage is 3 V, length-width (L:W) ratio is 2:1, and the silicon membrane thickness is 75 μm, the full scale output voltage of the pressure sensor is 15.50 mV at room temperature, and pressure sensitivity is 0.097 mV/kPa. When the supply voltage and L:W ratio are the same as the above, and the silicon membrane thickness is 45 μm, the full scale output voltage is 43.05 mV, and pressure sensitivity is 2.153 mV/kPa. Therefore, the sensor has higher sensitivity and good temperature characteristics compared to the traditional piezoresistive pressure sensor.

## 1. Introduction

At present, the various types of pressure sensors used include piezoresistive pressure sensors, capacitance pressure sensors, piezoelectric pressure sensors, resonator pressure sensors, and vacuum microelectronic pressure sensors, *etc.* [1–5]. In recent years, researchers have made use of the effects of the additional pressure P on channel resistance, gate capacitance C_ox_, threshold voltage V_T_, and channel carrier mobility μ_n_ (or μ_p_), to design and fabricate MOSFET pressure sensors [6–11]. For instance, Yan *et al.* [12] proposed a kind of MOSFET pressure sensor in 2001. Li *et al.* [13] designed and fabricated an integrated pressure sensor with a stress sensitive MOS operational amplifier in 2001. Zhang *et al.* [14] proposed a novel MEMS pressure sensor with MOSFET in 2008. Jachowicz *et al.* [15] of the Warsaw University of Technology fabricated a pressure sensitive field effect transistor (PSFET) in 2002. Fernández-Bolanõs *et al.* [16] reported the fabrication and electrical characterization of a novel pressure sensor based on a suspension-gate MOSFET(SG-MOSFET) using a new polyimide process. Garcia *et al.* [17] designed a pressure sensitive differential amplifier, whose sensitivity was 1.29 mV/kPa, and power was 3 μW. The main structure of the MOSFET pressure sensors is suspension gate structure, and differential structure of double tube, *etc.*

In order to improve the sensitive characteristics of the pressure sensor, and research the effects of supply voltage, membrane thickness and channel L:W ratio on the characteristics of the sensor, according to the piezoresistive effect, in this paper four p-MOSFETs using the nc-Si/c-Si heterojunction as source and drain, are designed and fabricated on a square silicon membrane by CMOS process and MEMS technology, and form a Wheatstone bridge with the four nc-Si/c-Si heterojunction MOSFETs channel resistances.

## 2. Basic Structure and Operation Principle

### 2.1. Basic Structure

**Figure 1.**
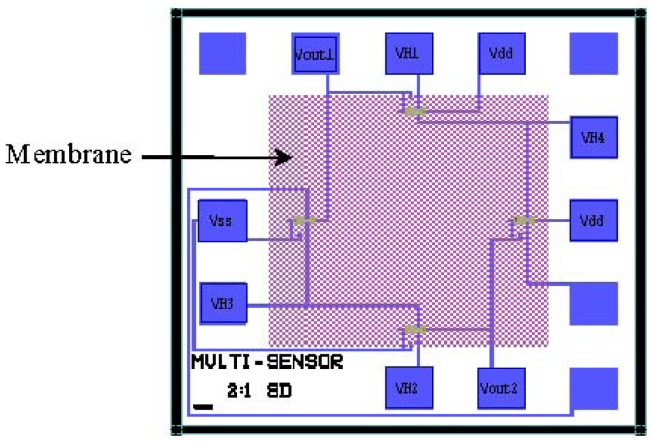
Mask layout of the nc-Si/c-Si heterojunction MOSFETs pressure sensor chip.

**Figure 2.**
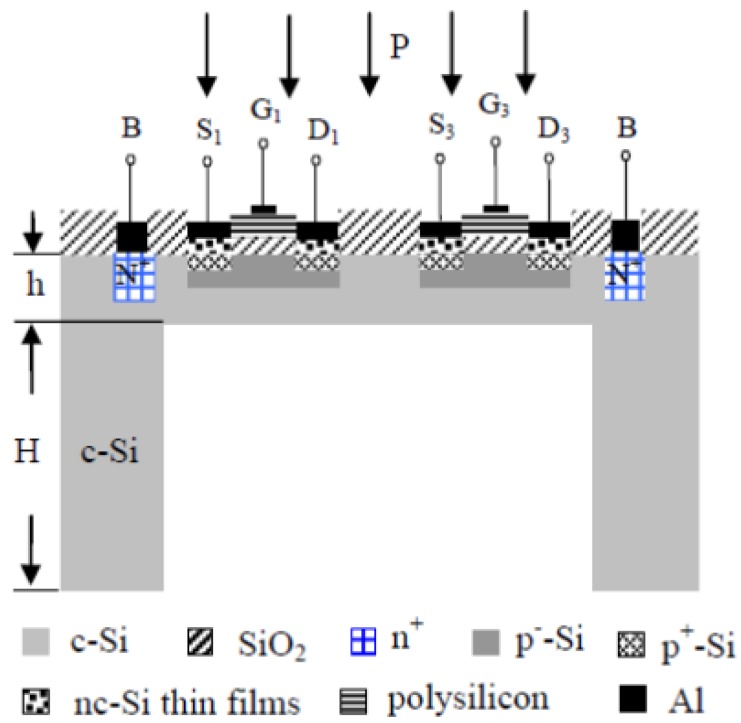
Cross-section of the nc-Si/c-Si heterojunction MOSFETs pressure sensor chip.

Figure 1 shows the chip layout of the MOSFETs pressure sensor, where the MOSFETs take the nc-Si/c-Si heterojunction as source and drain. In order to improve the sensitive characteristics of the pressure sensor, the nc-Si/c-Si heterojunction p-MOSFET is designed and fabricated on n-type <100> orientation single crystal silicon wafer, which has been polished on both sides by a CMOS process and MEMS technology, and a Wheatstone bridge is composed of four nc-Si/c-Si heterojunction MOSFETs channel resistances, so that the measurement of the additional pressure P can be achieved. Figure 2 shows the schematic cross-section of the nc-Si/c-Si heterojunction MOSFETs pressure sensor chip.

### 2.2. Operation Principle

**Figure 3.**
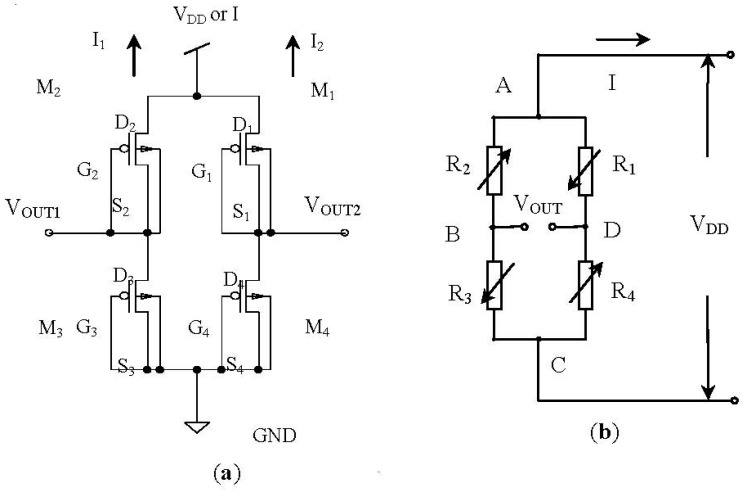
Operation principle of the nc-Si/c-Si heterojunction MOSFETs pressure sensor. (**a**) Wheatstone bridge; (**b**) Equivalent circuit.

Figure 3 shows operating principle schematic of the nc-Si/c-Si heterojunction MOSFETs pressure sensor. Figure 3(a) is the Wheatstone bridge structure composed of four nc-Si/c-Si heterojunction p-MOSFETs, which takes channel resistances R_1_, R_2_, R_3_ and R_4_ as piezoresistive resistances, Figure 3(b) is the equivalent circuit.

When the additional pressure P is zero, the output voltage V_OUT_ of the Wheatstone bridge is decided by four resistances R_1_, R_2_, R_3_, R_4_, and the constant bridge voltage V_DD_, as given by Equation (1):

(1) $$V_{\text{OUT}}=V_{\text{OUT}1}-V_{\text{OUT}2}=\frac{R_{1}R_{3}-R_{2}R_{4}}{\left(R_{1}+R_{2})(R_{3}+R_{4}\right)}V_{DD}$$

If *R*_1_ = *R*_2_ = *R*_3_ = *R*_4_ = *R*, then:

(2) $$V_{\text{OUT}}=V_{\text{OUT}1}–V_{\text{OUT}2}=0$$

When the additional pressure P isn't zero, channel resistances of the four nc-Si/c-Si heterojunction MOSFETs will change with the additional pressure P, V_OUT_ of the Wheatstone bridge is given by Equation (3):

(3) $$V_{\text{OUT}}=V_{\text{OUT}1}-V_{\text{OUT}2}=\frac{\left(R_{1}+\Delta R_{1})(R_{3}+\Delta R_{3})-(R_{2}+\Delta R_{2})(R_{4}+\Delta R_{4}\right)}{\left(R_{1}+\Delta R_{1}+R_{2}+\Delta R_{2})(R_{3}+\Delta R_{3}+R_{4}+\Delta R_{4}\right)}V_{DD}$$

If the variation of the channel resistances of the four nc-Si/c-Si heterojunction MOSFETs, are equal, as in the following Equation (4):

(4) $$\Delta R_{1}=-\Delta R_{2}=\Delta R_{3}=-\Delta R_{4}=\Delta R$$

Take Equation (4) into Equation (3), then:

(5) $$V_{\text{OUT}}=V_{\text{OUT}1}-V_{\text{OUT}2}=\frac{\Delta R}{R}V_{DD}$$

V_OUT_ of the Wheatstone bridge is proportional to the relative variation Δ*R/R* of the channel resistance and supply voltage V_DD_, respectively.

## 3. Fabrication

**Figure 4.**
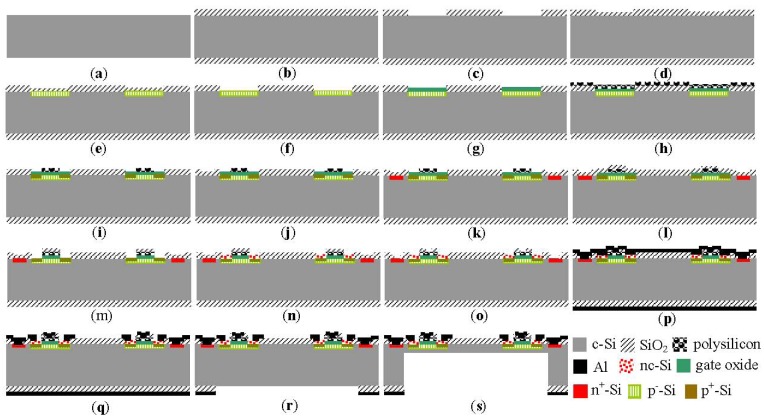
Fabrication technology process of the nc-Si/c-Si heterojunction MOSFETs pressure sensor chip.

A nc-Si/c-Si heterojunction MOSFETs pressure sensor is proposed in this paper, which adopts n-type <100> orientation single crystal silicon wafer with 4-inch high resistance (ρ > 100 Ω·cm), which has been polished on both sides and its thickness is 450 μm, Figure 4 shows the main fabrication technology process of the MOSFETs pressure sensor chip. Figure 4(a) single crystal silicon wafer; Figure 4(b) first oxidation, and SiO_2_ growth by a thermal oxidation process; Figure 4(c) first lithography, lithography active region window; Figure 4(d) second oxidation, and SiO_2_ growth by a thermal oxidation process in order to improve the uniformity of ion implantation; Figure 4(e) ion implantation, B ions implantation by ion implantation machine to obtain p-type doping, injection energy 40 KeV, injection dose of 6.0 × 10^13^; Figure 4(f) etching of SiO_2_ layer; Figure 4(g) third oxidation, the growth of gate oxide layer with thickness of 50 nm; Figure 4(h) growth of polysilicon gate by LPCVD and diffusion of phosphorus to the polysilicon gate; Figure 4(i) second lithography, lithographing polysilicon to form a polysilicon gate, and implantation of boron to form p-type doping for the source and drain of the MOSFET; Figure 4(j) third lithography; Figure 4(k) implantation of P, forming N^+^ substrate; Figure 4(l) oxidation of polysilicon, growth of SiO_2_ layer by synthesis of oxidation of H_2_ and O_2_; Figure 4(m) forth lithography to form the source and drain of the MOSFET; Figure 4(n) fifth lithography to grow the nc-Si thin film by LPCVD; Figure 4(o) sixth lithography to make lead holes; Figure 4(p) magnetron sputtering positively aluminum layer to the single crystal silicon wafer as aluminum electrode, and sputtering aluminum layer on the back, as passivation layer of ICP etching silicon; Figure 4(q) etching of C-type silicon cup window; Figure 4(r) eighth lithography, etching C-type silicon cup window; Figure 4(s) by adopting an ALCATEL 601E type ICP, deep groove etching to make nc-Si/c-Si heterojunction MOSFETs pressure sensor chip with 6 mm × 6 mm square silicon membranes of 75 μm and 45 μm thicknesses, respectively. The etch rate is about 4.5 μm/min.

**Figure 5.**
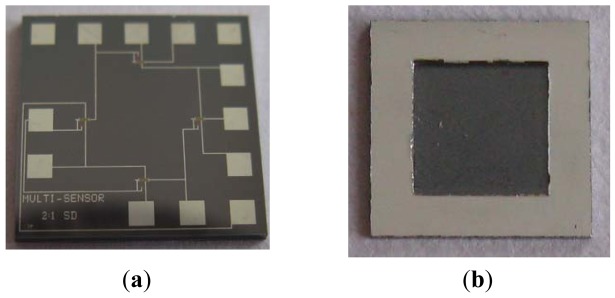
Photograph of the nc-Si/c-Si heterojunction MOSFETs pressure sensor chip with length-width ratio 2:1.

Figure 5 shows the photograph of the nc-Si/c-Si heterojunction MOSFETs pressure sensor chip with L:W ratio 2:1 proposed in this paper.

## 4. Experimental Results and Discussion

### 4.1. Piezoresistance Characteristics

**Figure 6.**
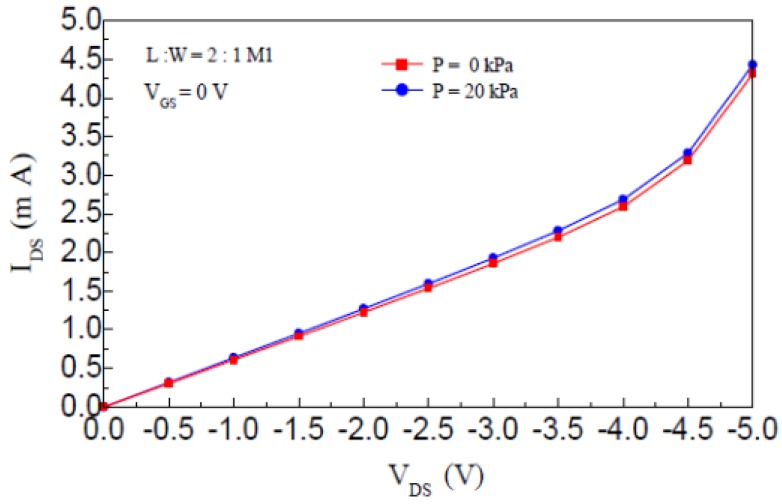
When P is constant, I_DS_-V_DS_ characteristics curves of the nc-Si/c-Si heterojunction MOSFETs.

Under the condition of an environmental temperature of 22 °C, and relative humidity of 15% RH, the piezoresistance characteristics of the M1 channel resistance of pressure sensor, which includes the nc-Si/c-Si heterojunction MOSFETs with L:W ratio 2:1 and square silicon membrane thickness 45 μm, is measured by a Mensor PCS400 Pressure Calibration System, Figure 6 shows the I_DS_-V_DS_ characteristic curves of the nc-Si/c-Si heterojunction MOSFETs when the additional pressure P is 0 kPa and 20 kPa, respectively.

### 4.2. Effect of Thickness of Silicon Membrane on Characteristics

When the test environment temperature is 22 °C, and relative humidity is 15% RH, calibration experiments of the nc-Si/c-Si heterojunction MOSFETs pressure sensor, which includes the nc-Si/c-Si heterojunction MOSFETs with L:W ratio 2:1 and square silicon membrane thickness 75 μm and 45 μm, respectively, are done using a Mensor PCS400 pressure calibration systems, HP34401A multimeter and BJ1790B power supply. The additional pressure range of the sensor with silicon membrane thickness 75 μm, are from 0 to 160 kPa, the additional pressure range of the sensor with silicon membrane thickness 45 μm, are from 0 to 20 kPa.

**Figure 7.**
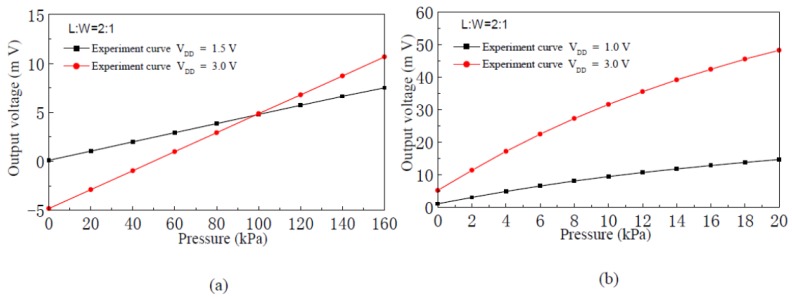
Input-output characteristics experimental curves of the nc-Si/c-Si heterojunction MOSFETs pressure sensor. (**a**) Membrane thickness 75 μm; (**b**) Membrane thickness 45 μm.

When the supply voltage V_DD_ is 1.0 V, 1.5 V and 3.0 V, respectively, Figure 7 shows the input-output characteristic experimental curves of the pressure sensor with channel L:W of 2:1 and silicon membrane thickness of 75 μm and 45 μm, respectively. The experimental results show that, when the silicon membrane thickness is constant, the full scale output voltage of the sensor is proportional to the supply voltage. On the condition that the supply voltage is 3.0 V, the full scale (160 kPa) output voltage of the sensor is 15.50 mV and the sensitivity of the pressure sensor is 0.097 mV/kPa when the silicon membrane thickness is 75 μm, and the full scale (20 kPa) output voltage of the sensor is 43.05 mV, and sensitivity of the pressure sensor is 2.153 mV/kPa when the silicon membrane thickness is 45 μm. The experimental results show that the sensitivity of the nc-Si/c-Si heterojunction MOSFETs pressure sensor is inversely proportional to the silicon membrane thickness.

**Figure 8.**
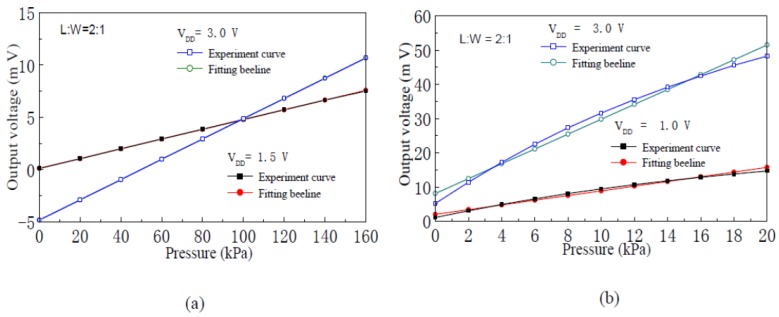
Input-output characteristics fitting beelines and experimental curves of the nc-Si/c-Si heterojunction MOSFETs pressure sensor. (**a**) Membrane thickness 75 μm; (**b**) Membrane thickness 45 μm.

Figure 8(a,b) shows fitting straight lines and experimental curves of the input-output characteristics of the pressure sensor with silicon membrane thicknesses of 75 μm and 45 μm, respectively. When the supply voltage is 3.0 V, linearity of the pressure sensor with silicon membrane thicknesses of 75 μm and 45 μm, is 0.130% F.S and 7.73% F.S, respectively.

**Figure 9.**
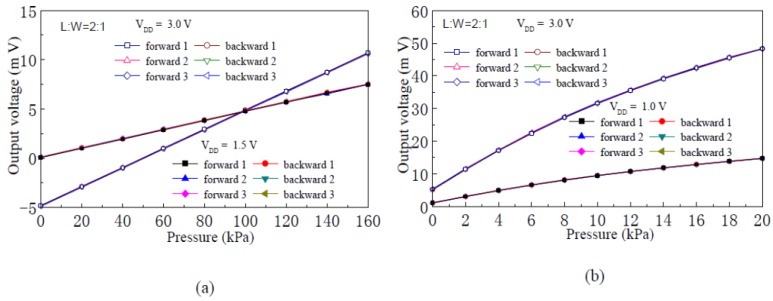
Repeatability experimental curves of the nc-Si/c-Si heterojunction MOSFETs pressure sensor. (**a**) Membrane thickness 75 μm; (**b**) Membrane thickness 45 μm.

Figure 9(a,b) shows repeatability experimental curves of the pressure sensor with silicon membrane thickness of 75 μm and 45 μm, respectively. When the supply voltage is 3.0 V, repeatability of the pressure sensor with silicon membrane thicknesses of 75 μm and 45 μm is 0.66% F.S and 5.42% F.S, respectively.

**Figure 10.**
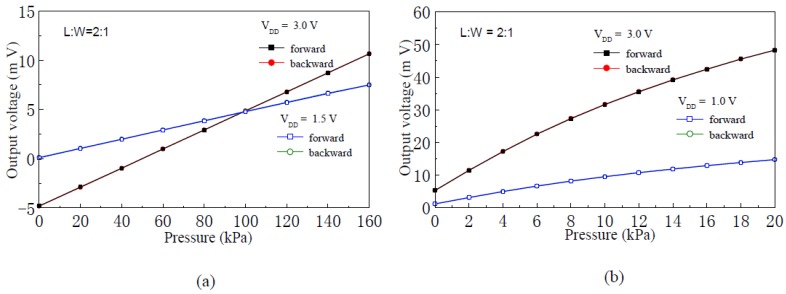
Hysteresis experimental curves of the nc-Si/c-Si heterojunction MOSFETs pressure sensor. (**a**) Membrane thickness 75 μm; (**b**) Membrane thickness 45 μm.

Figure 10(a,b) shows the hysteresis experimental curves of the pressure sensor with silicon membrane thicknesses of 75 μm and 45 μm, respectively. When the supply voltage is 3.0 V, hysteresis of the sensor with silicon membrane thicknesses of 75 μm and 45 μm, is 0.15% F.S and 2.74% F.S, respectively.

### 4.3. Effect of Length-Width Ratio of the MOSFET Channel on Characteristics

**Figure 11.**
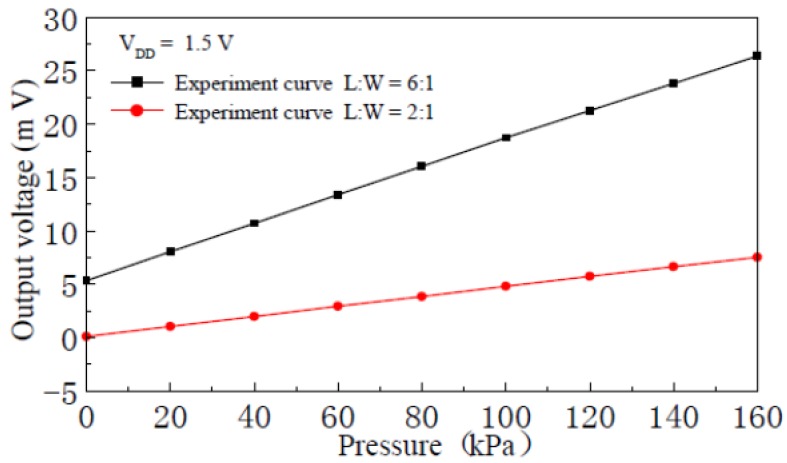
Input-output characteristics experimental curves of the nc-Si/c-Si heterojunction MOSFETs pressure sensor with different channel length-width ratios.

When the supply voltage is 1.5 V, Figure 11 shows the input-output characteristic experimental curves of the pressure sensor with the nc-Si/c-Si heterojunction MOSFETs, which has a silicon membrane thickness 75 μm and channel L:W ratios of 2:1 and 6:1. The experimental results show that when the supply voltage is constant, the sensitivity of the nc-Si/c-Si heterojunction MOSFETs pressure sensor is proportional to L:W ratio for the nc-Si/c-Si heterojunction MOSFET channel.

### 4.4. Temperature Characteristics

**Figure 12.**
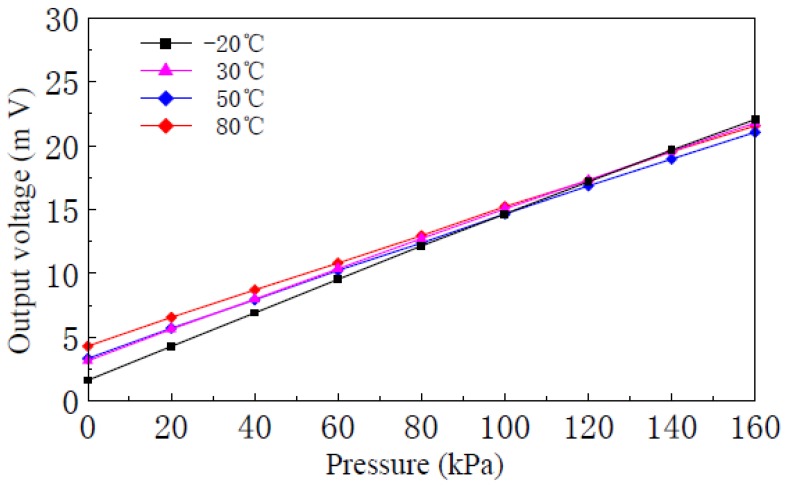
Input-output characteristics curves of the nc-Si/c-Si heterojunction MOSFETs pressure sensor at different temperatures.

High and low temperature experiments are done from −20 °C to 80 °C using a Shanghai Blue Leopard LGS-010C high and low temperature chamber. When the supply voltage is 1.5 V, Figure 12 shows different temperature input-output characteristics experimental curves of the nc-Si/c-Si heterojunction MOSFETs pressure sensor with channel L:W ratio of 6:1. Zero temperature coefficient *TCO*:

(6) $$\text{TCO}=\frac{V_{0}(T_{2})-V_{0}(T_{1})}{V_{0}(T_{1})(T_{2}-T_{1})}\times 100\%=16320\text{ppm}/{}^{\circ}C$$

where *V*_0_(*T*_1_) and *V*_0_(*T*_2_) are the zero outputs of the sensor when the temperature is T_1_ and T_2_, respectively. Sensitivity temperature coefficient *TCS*:

(7) $$TCS=\frac{\left[V_{F.S}(T_{2})-V_{0}(T_{2})]-[V_{F.S}(T_{1})-V_{0}(T_{1})\right]}{\left[V_{F.S}(T_{1})-V_{0}(T_{1})](T_{2}-T_{1}\right)}\times 100\%=-1550ppm/{}^{\circ}C$$

where *V*_F.S_(*T*_1_) and *V*_F.S_(*T*_2_) are the full scale outputs of the sensor when the temperature is T_1_ and T_2_, respectively.

## 5. Conclusions

In order to measure the additional pressure P, a MOSFET using a nc-Si/c-Si heterojunction as source and drain, is produced on high resistance single crystal silicon substrate with n-type <100> orientation by CMOS process and MEMS technology, and a Wheatstone bridge structure is composed of four nc-Si/c-Si heterojunction MOSFETs. Full scale output voltage of the pressure sensor is proportional to the supply voltage. When the silicon membrane thickness is 75 μm, full scale (160 kPa) output voltage of the pressure sensor is 15.50 mV and sensitivity is 0.097 mV/kPa when the supply voltage is 3.0 V. When the silicon membrane thickness is 45 μm, full scale (20 kPa) output voltage is 43.05 mV and sensitivity is 2.153 mV/kPa when the supply voltage is 3.0 V, so the sensitivity of the pressure sensor is inversely proportional to the silicon membrane thickness. When the silicon membrane thickness and supply voltage are constant, the sensitivity of the pressure sensor is proportional to the channel L:W ratio. When the temperature varies from −20 °C to 80 °C, temperature coefficients of zero point and sensitivity of the nc-Si/c-Si heterojunction MOSFETs pressure sensor with channel L:W ratio of 6:1, are 16,320 ppm/°C and −1,550 ppm/°C, respectively, so the nc-Si/c-Si heterojunction MOSFETs pressure sensor has higher sensitivity and a lower temperature coefficient compared to traditional piezoresistive pressure sensors.
